# Sexual Health of Haitian Immigrants in Southern Brazil: A Cross-Sectional Study

**DOI:** 10.5334/aogh.2666

**Published:** 2020-02-27

**Authors:** Kesta Saint-Val, Eliana Wendland

**Affiliations:** 1 Federal University of Health Sciences of Porto Alegre (UFSCPA), BR; 2 Department of Collective Health, Federal University of Health Sciences of Porto Alegre (UFSCPA), BR

## Abstract

**Background:**

Some specific groups, such as immigrants, are considered at significantly high risk of developing poor sexual health (SH), specifically in relation to sexually transmitted infections (STIs). However, despite the high number of Haitian immigrants living in southern Brazil, a study that assessed the SH of these immigrants has not been conducted yet.

**Objective:**

This study aimed to assess the sexual health of Haitian immigrants in southern Brazil.

**Methods:**

This was a cross-sectional study conducted in 201 adult Haitian immigrants of both sexes, living in southern Brazil. A self-applied questionnaire containing sociodemographic questions and SH and behaviors was used to collect data. Data were collected on paper or through online form.

**Findings:**

Of the 201 immigrants included in the study, the majority were men (58.06%). There was no difference in the mean age (30 years) between both sexes. More than half were married with a partner (a) (53.29%), and 69.89% had an average educational level. Although an association between the reported STIs and the variables considered as risk factors or behaviors for STIs was not reported, women reported a frequency of 33.80% for self-declared active STIs and a frequency of 66.20% for lifelong STIs; these frequencies are highly superior in women compared to men. Additionally, 55.33% of women also reported not using a condom in their last sexual intercourse, and 35.10% reported changing their sexual behaviors after immigrating.

**Conclusions:**

The high frequency of STIs reported by Haitian immigrant women indicates the need to implement STI tracking strategies in that population. This study can assist in the development of comprehensive healthcare policies for Haitian immigrants.

## Introduction

Migrations have significant effects on the immigrants’ general health and sexual health (SH) considering that every foreign country has different sexual practices and beliefs [1], and that these immigrants have to adapt to a new healthcare system. Hence, some diseases, specifically infectious ones, such as sexually transmitted infections (STIs), human immunodeficiency virus (HIV) infection/acquired immunodeficiency syndrome (AIDS), and hepatitis, are more easily transmitted in immigrants than in non-immigrants [2]. However, generally, knowledge about the epidemiological profile of STIs in the immigrant population is insufficient considering that these immigrants do not have access to healthcare services, which contributes to the under-notification of STIs [3].

According to the World Health Organization (WHO), deaths and disabilities related to the problems of SH represent one-third of the world’s morbidity burden among women of reproductive age (15–44 years old) and almost 20% of the overall disease morbidity burden [4]. The WHO also emphasized that more than one million individuals acquire an STI daily worldwide [3,5]. In Haiti, morbidity is dominated by HIV/AIDS-related infections, particularly in women, with a prevalence of 1.2% [6]. It is estimated that 85% of HIV-positive adults in the Caribbean live in Haiti [7].

In Brazil, chlamydia and gonorrhea are the highest rates of curable infections in the sexually active population, with 1,967,200 and 1,541,800 cases, respectively. In the southern region of the country, the AIDS detection rate in the last 10 years has remained stable, with an average of 31.1 cases per 100,000 inhabitants [8].

Brazil has more than 73,094 Haitian immigrants [9], of whom between 2,500 and 7,000 live in the State of Rio Grande do Sul [10]. Despite this, the epidemiological profiles of STIs and other characteristics related to the SH of Haitians are unknown in Brazil, considering that a study that assessed the SH of Haitians has not been conducted yet.

This study aimed to evaluate the SH of the Haitian immigrant population living in the State of Rio Grande do Sul, identifying in a self-reported manner the prevalence of STIs, factors associated with STI presence, and changes in sexual behaviors related to migration.

## Materials and Methods

### Description of the study

This was a cross-sectional study based on the data on sexual health and immigration. These data were derived from the questionnaire that was administered to the participants. The study also combined the elements of quantitative-qualitative approaches, describing the variables and characterizing the variables’ association with the classified variables.

### Population and study sample

The immigrants of both sexes from the Republic of Haiti living in Brazil represented the study’s population. A sample of 201 Haitians living in the State of Rio Grande do Sul participated in the study.

### Inclusion criteria

- Individuals who were native immigrants from Haiti
- Individuals aged older than 18 years
- Individuals living in the State of Rio Grande do Sul for at least 6 months
- Individuals who were able to read and understand French or Portuguese.

### Places and method of recruitment

The data were collected in Portuguese classes for immigrants, humanitarian centers, churches of the Haitian community, and via an online invitation. These different environments were frequently visited by Haitian immigrants. Before the start of the study, a previous contact was established with individuals supervising these places via email and phone call, or through personal contact with these individuals for data collection planning. During the first contact, we briefly presented the purpose and importance of the study and the method of recruitment of the participants.

Regarding the sampling technique, “snowball” sampling was used. In this technique, some individuals were identified by the researcher, and they were classified as *seeds* (wave 0). These individuals were asked to initiate contact with other individuals, classified as *wave 1*. This process was repeated successively until the total number of the sample was finally obtained. It was estimated that 390 individuals were required to detect a prevalence of 15%, with an odds ratio of 2.1, a power of 80%, and a level of significance of 95% [11]. Of the estimated sample, 201 individuals from different cities agreed to participate in the study. Considering the daily situation of the immigrants, with the fear of possibly passing personal information to other individuals and with the immigrants’ insufficient time, this study was not able to investigate the entire immigrant population, taking care of the representativeness of the sample in relation to the target population to establish a characteristic sample. Hence, 185 immigrants from Porto Alegre, 5 from Caxias do Sul, 10 from Canoas and Novo Hamburg, and 1 from Lajeado participated in the survey.

### Instrument and data collection

In developing the questionnaire, the main variables were defined, which facilitated the formulation of the questions. Some of the questions in the questionnaire were derived from the 2010 Census of the Brazilian Institute of Geography and Statistics, comprising close- and open-ended questions about the immigrants’ sociodemographic data, reproductive health, and sexual behaviors. These questions were divided into five sections. The first four sections were related to immigrants’ sociodemographic data and lifestyle-related behaviors (alcohol intake, smoking status, illicit drug use, and physical activity). The last section referred to the SH and reproduction (SHR) area, containing questions about the presence of current and past STI and age of first sexual intercourse; number of partners in the last 3, 6, and 12 months; history of prostitution; changes in sexual behaviors due to immigration; and history of HIV infection.

The original questionnaire (Portuguese) and its translation into French language were evaluated by a professional with experience in the SHR area. A pilot study was conducted to determine any challenges encountered in the study, and the study’s methodology was modified. Participants’ identification was withdrawn, and interviews were incorporated, aiming at the privacy of the interviewees and ensuring the confidentiality of the participants’ information. The recruitment took place for 6 months, from August 2018 to January 2019. Once in the recruitment sites, the Free and Clear Consent Term (FCCT) was distributed to the participants, with the contents and purposes of the study comprehensively explained to the participants. Additionally, information regarding STIs was placed in a folder, and male and female condoms were given to the participants as a form of appreciation for their participation in the study.

It should be noted that the FCCT was also written in two languages (Portuguese and French) so that the participants could comprehensively understand the objectives of the study. After reading the document, if an individual agreed to participate in the study, he/she had to sign the FCCT presented on two forms: one given to the participant and the other kept by the researcher. The same considerations were observed for online collection.

### Statistical analysis

All analyses were performed using the R software 3.5.1. The chi-squared test (X^2^) and Fisher’s exact test were used to test the significance of the differences between proportions, and the t-test was used to evaluate the differences between averages. Initially, the univariate analysis was performed, estimating the association between the reported STIs and the following variables investigated: age, sex, condom use, marital status, period of residence, and number of partners in the last 6 months. Subsequently, some of these variables were included in a modified Poisson model (robust variance) to estimate the prevalence ratio. Prevalence rates were calculated with a 95% confidence interval, and P < 0.05 values were considered statistically significant.

### Ethical considerations

This study was conducted in accordance with the National Health Council’s Resolution 466 of December 12, 2012 (BRAZIL, 2013), offering the maximum benefits and minimum risks and harm to the participants involved. Considering the ethical precepts, the participants voluntarily participated in the study. This study was approved by the Research Ethics Committee of the Federal University of Health Sciences of Porto Alegre (Sight n. 2.660.997). All participants signed the FCCT after they were informed about the contents and purposes of the study.

## Results

Among the 201 individuals who participated in the survey, 58.06% were men. There was no significant difference between the mean age of men (31 years) and women (30 years) (p = 0.04). Moreover, a higher proportion of women in the younger age group and a higher proportion of men in the older age group were observed. Women and men were similar regarding their marital status. A higher proportion of men were employed (78.50%). Although there was no difference on the educational level between sexes, most of the participants were in high school level of education, and 28% of men presented with higher educational level. The vast majority of participants received less than R$ 1.874,00 per month, with men presenting with higher income than women. Moreover, approximately 50% of women had no income (Table 1).

**Table 1:** Sociodemographic characteristics according to sex of Haitian immigrants living in Rio Grande do Sul, Brazil, 2018.

Characteristics	Total (n = 201)		Male (n = 108)		Female (n = 78)		p*

	n	%	n	%	N	%	

**Age (years old)**							0.04
18–24	35	20.96	12	12.90	23	31.50	
25–44	89	53.29	52	55.90	37	50.70	
45–64	38	23.35	28	30.10	10	13.70	
More than 65	4	2.40	1	1.10	3	4.10	
**Education level**							0.20
Complete Fundamental education/incomplete	23	12.23	13	12.00	10	12.80	
Complete Medium education/incomplete	122	64.89	65	60.20	55	70.50	
Complete High education/incomplete	43	22.87	30	27.80	13	16.70	
**Marital situation**							0.63
Single	41	21.81	25	23.10	15	19.20	
Dating	40	21.28	20	18.50	20	2.60	
Married	101	53.72	60	55.60	40	51.30	
Separated/divorced	6	3.19	3	2.80	3	3.80	
**Occupation**							<0.005
Employed	116	62.03	84	78.50	31	39.70	
Unemployed	71	37.97	23	21.50	47	60.30	
**Monthly income R$**							<0.005
<937,00	48	26.97	32	30.50	15	21.10	
937,00–1874,00	59	33.15	41	39.00	17	23.90	
1875,00–4685,00	15	8.43	15	14.30	0	0	
Without income	50	28.09	13	12.40	37	52.10	
Not informed	6	3.37	4	3.80	2	2.80	
**Time of residence in Brazil**							0.32
<1 year	31	18.79	14	15.70	15	20.80	
1–<2 year	29	17.58	16	18.00	13	18.10	
2–<3 years	47	28.48	19	21.30	27	37.50	
≥3 years	57	34.55	40	44.90	17	23.60	

* Chi-squared test p value. The sum of the values varies according to the variable due to the missing data.

Regarding sexual partners, more than half (57.14%) had a sexual partner during the last 3 months, and only 3.01% had more than two sexual partners (all men). More than half (63.19%) of the participants reported to have a regular partner during the last 6 months. Most (64.9%) participants referred to using a contraceptive method, with the use of condom being the most common (42.79%). However, only half (49.16%) of them reported using condoms in the last sexual intercourse. Moreover, women used contraceptive methods less than men. The most mentioned sexual practices were vaginal penetration, hugging, and oral sex (64.18%, 47.26%, and 24.38%, respectively). Only 2.33% of the participants reported having sex with partners of both sexes, and 1.73% reported having sex with partners of the same sex (all men). The two main meeting places of couples today were restaurants/parties (31.43%) and schools (28.57%) (Table 2).

**Table 2:** Risk behaviors for sexually transmitted infection according to sex in Haitian immigrants living in the State of Rio Grande do Sul, Brazil, 2018.

Characteristics	Total (n = 201)		Male (n =108)		Female (n =78)		p*

	N	%	n	%	N	%	

**Number of partners in the last 6 months**							0.01
None	29	21.80	17	23.00	12	20.70	
1	76	57.14	34	45.90	41	70.70	
2	22	16.54	17	23.00	5	8.60	
>2	4	3.01	4	5.40	0	0	
**Contraceptive methods**							0.19
Yes	116	64.09	60	58.80	55	71.40	
No	54	29.83	34	33.30	19	24.70	
Not informed	11	6.08	8	7.80	3	3.90	
**Condom use in the last sexual intercourse**							0.42
Yes	76	42.46	47	46.50	28	36.80	
No	91	50.84	48	47.50	42	55.30	
Not informed	12	6.70	6	5.90	6	6.00	
**Practices during the sexual act**							
Hugs and kisses	95	47.26	51	47.20	43	55.10	0.28
caresses and/or masturbation	21	10.45	13	12.01	8	10.30	0.70
Oral sex	49	24.38	25	23.10	24	30.80	0.24
Anal sex	12	5.97	5	4.60	7	9.00	0.23
Others practices	2	1	1	0.90	1	1.30	0.81
Vaginal sex	129	64.18	71	65.70	57	73.10	0.28
**Partner sex (last 6 months)**							
Of the same gender	3	1.74	3	3.10	0	0	
Of the opposite gender	153	88.95	84	85.70	68	93.30	
Both genders	4	2.33	4	4.10	0	0	
Not informed	12	6.98	7	7.10	5	6.80	
**Type of sexual partner (last 6 months)**							<0.005
Regular	103	63.19	43	46.70	59	84.30	
Not regular without payment	34	20.86	31	33.70	3	4.30	
Business	1	0.61	1	1.10	0	0	
Not informed	25	15.34	17	18.50	8	11.40	
**Meeting place of the current partner**							0.12
Family house	11	6.29	7	7.20	4	5.30	
Friend’s house	12	6.86	11	11.30	1	1.30	
School	50	28.57	21	21.60	28	36.80	
Work	14	8.00	8	8.20	6	7.90	
Travel	5	2.86	2	2.10	3	3.90	
Churches	18	10.29	9	9.30	9	11.80	
Restaurants/parties	55	31.43	30	30.90	23	30.30	
Public places	9	5.14	7	7.20	2	2.60	
Online	1	0.57	1	1	0	0	

* Chi-squared test p value. The sum of the values varies according to the variable due to the missing data.

**Figure 1:**
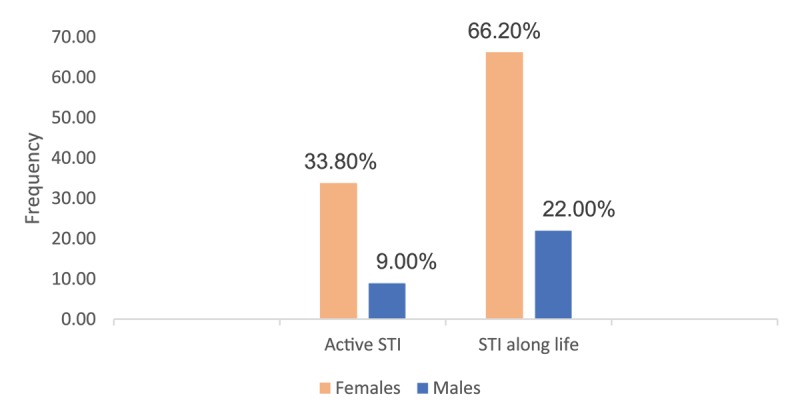
Frequency of self-declared active and lifelong sexual infection among Haitian immigrants according to sex in Rio Grande do Sul, Brazil, 2018.

The presence of signs and symptoms during sexual intercourse was also reported more frequently by women, with pain (26.9%) and burning (15.4%) sensation being the two main symptoms reported, compared to men (1.9% and 3.7%, respectively) (Table 3). Moreover, 66.20% of women and 22.00% of men declared having had STI at least once in their lifetime, and 38.80% of these women and 9% of these men declared presenting an STI at the time of the interview (Figure 1). It should be emphasized that trichomoniasis was the most common STI reported by women.

**Table 3:** Report of human immunodeficiency virus infection/sexually transmitted infection and changes in the sexual behavior of Haitian immigrants in the State of Rio Grande do Sul, Brazil, 2018.

Characteristics	Total (n = 201)		Male (n =108)		Female (n =78)		p*

	N	%	n	%	N	%	

**STI diagnosis**							<0.005
No	83	46.37	58	58.00	24	31.20	
Yes, but there’s no more	38	21.23	13	13.00	25	32.50	
Yes, I still have	12	6.70	2	2.00	10	13.00	
Yes, but I don’t know if I still have	23	12.85	7	7.00	16	20.80	
Not informed	6	3.35	5	5.00	1	1.30	
Unknown	17	9.50	15	15.00	1	1.30	
**Symptoms and Signs During Sexual Act**							
Pain	23	11.44	2	1.90	21	26.90	<0.005
Bleed	1	0.50	1	0.90	0	0	0.39
Stinging	16	7.96	4	3.70	12	15.40	0.01
**He/she has had an HIV test**							0.90
Yes	141	76.63	79	76.00	61	78.20	
No	40	21.74	23	22.10	16	20.50	
Not informed	3	1.63	2	1.90	1	1.30	
**Changes in sexual habits**							0.004
Yes	43	24.60	16	16.30	27	35.10	
Not	132	75.40	82	83.70	50	64.90	

* Chi-squared test p value; STI, sexually transmitted infections. The sum of the values varies according to the variable due to the missing data.

Regarding the HIV test, 76.63% of the participants reported undergoing the HIV test at some point in their lives. However, for participants who knew the results of their HIV test, none of them have declared a positive result. Regarding the habits during sexual intercourse, 24.72% of the interviewees reported changes in their sexual behaviors (Table 3).

Although there was a difference in the proportion between the sexes regarding the presence of self-declared active STIs (Table 3), none of the factors studied were associated with the presence of STIs considering that the prevalence ratio was calculated using the modified Poisson regression model (Table 4).

**Table 4:** Analysis of sociodemographic factors and risk behaviors associated with sexually transmitted infection prevalence according to the reports of Haitian immigrants in Rio Grande do Sul, Brazil, 2018.

Variable	Prevalence ratio	95% CI

**Age (years)**		
18–24	1	–
25–39	0.92	0.63–1.36
40 or more	1.02	0.62–1.66
**Gender**		
Female	1	–
Male	0.90	0.67–1.22
**Condom use in the last sexual intercourse**		
Yes	1	–
No	1.20	0.89–1.63
**Marital situation**		
Single	1	–
Dating	1.04	0.67–1.64
Married	1.12	0.78–1.65
Separated/divorced	1.56	0.64–3.29
**Education level**		
Complete Fundamental education/incomplete	1	–
Complete Medium education/incomplete	0.89	0.59–1.41
Complete High education/incomplete	0.74	0.46–1.24
**Time of residence in Brazil**		
<2 years	1	–
2 < 4 years	1.08	0.77–1.52
4 years or more	0.86	0.52–1.38
**Partner sex (last 6 months)**		
None	1	–
1	0.98	0.65–1.63
2	0.95	0.50–1.74
>2	0.76	0.34–1.54

* Chi-squared test p value. The sum of the values varies according to the variable due to the missing data.

## Discussions

Recently, Haitian men more frequently immigrate to Brazil compared to Haitian women [12]. Women have a higher frequency of self-declared lifelong active STIs than men. Most of the participants refer to having a low number of sexual partners and fixed partnerships, and a low frequency of respondents have performed sexual intercourse with same-sex individuals. None of the interviewees reported having HIV, although most of the participants had already undergone the test. This is the first study to address the SH of Haitian immigrants in Brazil and one of the few studies addressing the SH of Haitian immigrants in Brazil in the literature.

A survey conducted in Chapecó region, which aimed to evaluate the epidemiological profile of Haitian immigrants, consisted of 573 individuals (men, 54.1% [12], a proportion lower than that found in the study conducted in Rio Grande do Sul). Although the increase in the Haitian female population has been reported, the proportion of men who participated in the previous studies was significantly higher compared to women, a result consistent in the present study, suggesting that researchers have difficulty accessing the female population. However, the highest proportion of men could be explained by the fact that they first immigrate before women to look for work and a place of residence, eventually bringing their wives and children when they are already stable [13,14] Most immigrants have a good educational level, which has been confirmed in two other studies. The first one, which was conducted in Chapecó, reveals that most Haitians have completed high school or second grade [12]. In the second study, Da Silva reported that most Haitian immigrants have studied for more than 10 years, which is equivalent to high school educational level in Brazil [13].

Considering that studies assessing the SH of immigrants in Brazil have not been conducted yet, conducting compare studies is relatively difficult. However, a study comprising another population was conducted. According to a study [15] conducted in the triple border of the Amazon, Brazil, in which interviews and STI/HIV tests were used, the following STIs were observed in men and women: gonorrhea (1.1% and 0.3%, respectively), chlamydia (1.4% and 4.8%, respectively), active syphilis (3.2% and 2.6%, respectively), hepatitis C virus (0.7% and 0.7%, respectively), and HIV infection (1.4% and 0.0%, respectively). According to the study by Norris et al., HIV and syphilis screening tests were also performed, with a prevalence of HIV and syphilis of 9% and 12% in men and 6% and 11% in women, respectively [16]. The results of both the previous studies are inconsistent with the result of the present study. These differences are attributed to the significant differences in the population studied, with a widespread HIV epidemic in African countries and some Caribbean countries [17,18]. However, a study on migration and women’s health revealed that the health of immigrants has significantly worsened [19] considering that they are significantly discriminated as women and as immigrants [20,21,22,23]. Additionally, they are significantly at risk of sexual abuse, rape, and violence, specifically when they are staying in refugee camps [19,24], which may explain the higher frequencies of STIs observed.

Regarding sexual behaviors, although condom use has not been associated with the presence of STI, it is important to emphasize that more men reported using condoms than women. This fact is consistent with the study conducted by Martins et al. in Brazil that assessed the prevalence of STI, demonstrating the lack of condom use in women [25]. It has been concluded that women frequently have no protection and choices regarding their sexual practices because they submit themselves to their partners; thus, they are highly exposed to different types of transmitted diseases [25]. A common example is women living in developing countries who submit themselves to their partners during marital sexual intercourse, making them vulnerable to STI/HIV infection [26].

The vast majority of Haitian immigrants reported having only one sexual partner in the last 6 months. They also reported having heterosexual intercourse, with vaginal penetration as the most common sexual practice. These data are found in the traditional literature. Furthermore, considering that the frequency of STI was significantly high in this study, sexual intercourse with several partners and homosexual relationships are generally considered as risk factors for the transmission of STI/HIV [27]. Although these data were also found in the studies of Benzaken et al. and Borges, the first study conducted in the Amazon reported that only 38.5% of the individuals who participated in the study have reported condom use [15]. The second study consisted of immigrants from sub-Saharan Africa living in Lisbon and showed [28] that most immigrants declared themselves as heterosexual.

It is noteworthy that, in addition to the factors previously described, the study also found that 35.10% of immigrant women reported changes in their sexual behaviors. Specifically, they reported having increased sexual libido since they immigrated to Brazil. A study conducted in Turkey [29] confirmed this result, which seems to be frequently observed in studies conducted with individuals immigrating to Europe. The author explains that the change in social relations found in the West mainly affects immigrant women. A migrating woman feels significantly secured not only because she earns money and becomes financially independent but also because she is now living away from her native village, which sometimes controls an individual’s morality [29]. Hence, a migrating woman has a lower stress level, consequently increasing her sexual libido. However, this phenomenon has corresponding risk, known as acculturation, which has been studied in several immigrant groups [30,31].

## Limitations and Strengths

Some important limitations of the present study should be highlighted. There was a significant difficulty in accessing Haitian immigrants considering their insufficient knowledge about the existence of population research and their fear of participation because of the belief that the study might cause problems regarding their stay in the country; hence, this study has a small sample size. Despite the estimated 2.5 to 7,000 Haitian immigrants in Rio Grande do Sul, only 201 participants were included in the study. It should also be emphasized that SH is a controversial subject for the population studied. Therefore, the sensitivity and discomfort that the participants felt when answering the questions negatively affected their participation in the study. As a result, potential participants were provided with sufficient time in deciding whether they would like to participate in the study. This might lead to classification bias because we cannot exclude that individuals with active infection have not reported the presence of infection or were unaware of their condition due to insufficient healthcare access were. Although immigrants can access the Brazilian public healthcare services, cultural barriers such as language and culture may negatively affect their healthcare access and outcomes.

This study is the first to address the topic of sexual health in Haitian immigrants. Through this study, SH actions in immigrants—for example, strengthening the orientation for condom use as a contraceptive method—can be implemented. This study also serves as a reference in further conducting several studies that perform rapid diagnostic HIV/STI testing in this specific population in Brazil.

## Conclusion

In conclusion, this study shows that a higher frequency of STIs is observed in Haitian female immigrants compared to Haitian male immigrants. However, considering that several risk factors for STIs were determined in this study due to the participants’ significant differences, further studies with larger sample sizes are required to accurately determine the risk factors associated with the prevalence of STI in this vulnerable group, and studies incorporating HIV/STI tests to determine the presence of STI are also necessary.
